# The Changes in Plasmalogens: Chemical Diversity and Nutritional Implications—A Narrative Review

**DOI:** 10.3390/nu17223497

**Published:** 2025-11-07

**Authors:** Zhen Chen, Chen Dong, Lin Chen, Meiling Song, Xinxin Zhou, Depeng Lv, Quancai Li

**Affiliations:** 1 School of Pharmacy, Jiangsu University, Zhenjiang 212013, China; dongchen@stmail.ujs.edu.cn (C.D.); chenlin@stmail.ujs.edu.cn (L.C.); songmeiling@stmail.ujs.edu.cn (M.S.); zhouxinxin@stmail.ujs.edu.cn (X.Z.); 2 Marine Biomedical Research Institute of Qingdao, Qingdao 266071, China; lvdepeng@ouc.edu.cn; 3 Key Laboratory of Marine Drugs, Ministry of Education, Shandong Provincial Key Laboratory of Glycoscience and Glycoengineering, School of Medicine and Pharmacy, Ocean University of China, Qingdao 266003, China

**Keywords:** dietary phospholipids, oxidation, degradation, molecular species, lipid metabolism, functional food ingredients

## Abstract

Plasmalogens, as natural dietary lipids, are a unique class of glycerophospholipids with distinct structural and functional properties. They are unstable due to the vinyl ether linkage and the unsaturated fatty chains. Hence, plasmalogen changes are closely connected to their beneficial bioactivities and health-related applications. This narrative review focuses on their structural modifications, particularly oxidation of the vinyl-ether and sn-2 acyl chains, enzymatic degradation, and molecular remodeling. The oxidative susceptibility of plasmalogens renders them particularly vulnerable under inflammatory or oxidative stress, contributing to a measurable reduction in total plasmalogen content. Plasmalogen deficiency has been observed in various diseases and applied in clinical applications, including physiological and a variety of pathological conditions. Moreover, plasmalogens have been recognized as not only disease biomarkers but also therapeutic targets. In addition, recent findings in nutrition were discussed, aiming to find that underutilized animal byproducts and microbial lipids are promising new sources of plasmalogens. To conclude, it is crucial to establish practical dynamic monitoring systems of plasmalogens for health promotion and disease prevention interventions. Integrating biochemical pathways, clinical diagnosis, and nutritional interventions remains to be clarified.

## 1. Introduction

**Figure 1:**
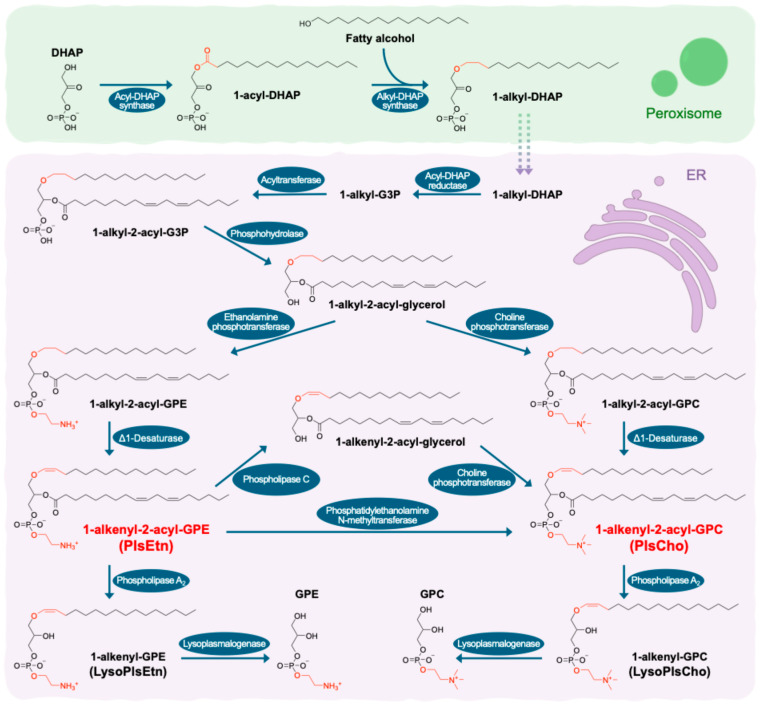
Structures, biosynthesis, and metabolism of ethanolamine (PlsEtn) and choline (PlsCho) plasmalogens (summarized based on references [4,5,6]), taking PlsEtn p16:0/18:2 *n*-6 and PlsCho p16:0/18:2 *n*-6 as examples. DHAP, dihydroxyacetone phosphate; G3P, glycerol-3-phosphate; GPE, glycerophosphoethanolamine; GPC, glycerophosphocholine; ER, endoplasmic reticulum.

Plasmalogens are a distinct type of membrane glycerophospholipids characterized by a fatty alcohol chain with a vinyl ether (O-alk-1′-enyl) bond at the sn-1 position, a commonly unsaturated fatty acyl chain at the sn-2 position, and the headgroups attached to the glycerol backbone. The sn-1 chain is typically derived from palmitic, stearic, or oleic acid, while the sn-2 fatty acyl is commonly unsaturated, such as oleoyl, linoleoyl, arachidonoyl, eicosapentaenoyl, docosahexaenoyl, and others [1]. Common headgroups of plasmalogens are choline (PlsCho) and ethanolamine (PlsEtn) [2] (Figure 1); in addition, there are also less common ones like serine and inositol [3]. The diversity of the fatty chain composition at the sn-1 and sn-2 positions, as well as the headgroups, reflects the complex enzymatic pathways and substrate specificities involved in plasmalogen biosynthesis, which plays a critical role in determining the physical properties of cell membranes and the functional diversity of these lipids in cellular processes.

Plasmalogen biosynthesis begins in the peroxisome, starting with the synthesis of the precursor dihydroxyacetone phosphate (DHAP), followed by the acylation of DHAP to form 1-acyl-DHAP, which is catalyzed by acyl-DHAP synthase. The distinctive feature of plasmalogens, the vinyl-ether bond at the sn-1 position, is introduced by a subsequent enzyme, alkyl-DHAP synthase, which replaces the fatty acyl group with a fatty alcohol, to form 1-alkyl-DHAP. This intermediate is transported from the peroxisome to the endoplasmic reticulum (ER) and reduced to 1-alkyl-glycerol-3-phosphate (1-alkyl-G3P), a process facilitated by acyl-DHAP reductase. Next, modifications such as acylation and hydrolysis occur in the ER, followed by forming the phosphodiester bond linking the glycerol backbone to the headgroup. These steps, catalyzed by ethanolamine phosphotransferase (EPT) or choline phosphotransferase (CPT), finalize the synthesis of PlsEtn or PlsCho, respectively, which are the predominant types. PlsEtn can also be converted to PlsCho via phosphatidylethanolamine *N*-methyltransferase (PEMT) or the phospholipase C (PLC)-CPT pathway. Both PlsEtn and PlsCho can be hydrolyzed to produce lysoplasmalogens and, subsequently, glycerophosphoethanolamine (GPE) and glycerophosphocholine (GPC), respectively (Figure 1) [4,5,6].

Plasmalogens are present in animals and some microorganisms. In mammals (including humans), they are necessary structural phospholipids in cell membranes, specifically abundant in the brain, heart, kidney, lung, and skeletal muscle. Plasmalogens play crucial roles in a wide range of physiological processes and conditions, such as maintaining cellular integrity, mediating cell membrane dynamics, participating in signal transduction, and others. At the same time, plasmalogens have been associated with a wide range of dysfunctions and diseases, including cell membrane alterations, fatty alcohol accumulation, neurodegenerative diseases, metabolic syndromes, respiratory diseases, and other lipid metabolic disorders [5,7,8,9,10].

Given the significant roles of plasmalogens in health and their connection to various disorders, it is necessary to explore how these molecules change and vary. Hence, the present narrative review aims to summarize the structural changes in plasmalogens, their variations in humans influenced by different factors, and their external sources for nutritional supplements. The current limitations, challenges, and future perspectives are also discussed.

## 2. Materials and Methods

### 2.1. Literature Search Strategy

Comprehensive data collection was conducted to explore different aspects of plasmalogen changes in clinical nutrition and health. Relevant articles were identified through an electronic search of the PubMed database. The search utilized a combination of terms related to plasmalogens and their broader contexts. The language filter was set to English, and the publication date filter was set from January 2005 to the present.

The following search terms were used: ((“plasmalogen” [tiab] OR “plasmenylcholine” [tiab] OR “plasmenylethanolamine” [tiab] OR “PlsCho” [tiab] OR “PlsEtn” [tiab] OR “ether lipid” [tiab] OR “vinyl-ether lipid” [tiab]) AND ((“biosynthesis” [tiab] OR “metabolism” [tiab] OR “catabolism” [tiab] OR “pathway” [tiab] OR “peroxisome” [tiab] OR “degradation” [tiab]) OR (“antioxidant” [tiab] OR “oxidative stress” [tiab] OR “neuroprotection” [tiab] OR “inflammation” [tiab] OR “ferroptosis” [tiab] OR “membrane fluidity” [tiab]) OR (“Alzheimer’s disease”[tiab] OR “Parkinson’s disease” [tiab] OR “dementia” [tiab] OR “neurodegeneration”[tiab] OR “multiple sclerosis” [tiab] OR “cardiovascular disease” [tiab] OR “atherosclerosis” [tiab] OR “metabolic syndrome” [tiab]) OR (“diet” [tiab] OR “dietary” [tiab] OR “supplement” [tiab] OR “clinical trial” [tiab]) OR (“lipidomics” [tiab] OR “mass spectrometry” [tiab] OR “LC-MS” [tiab] OR “GC-MS” [tiab]))) AND English [la] AND 2005:2024 [dp]. The initial search yielded 901 records. In addition, the reference lists of retrieved literature were manually screened (“snowballing”) using the search engine Google Scholar to identify potentially associated research.

### 2.2. Study Selection and Eligibility Criteria

After removing duplicates, titles and abstracts were screened for relevance. Subsequently, full-text articles were assessed against predefined eligibility criteria. Studies were included if they: (1) investigated qualitative or quantitative changes in plasmalogens; (2) were related to their chemical, biological, clinical, or nutritional purposes; and (3) were published in English as original research articles or reviews, while studies were excluded if they were: (1) patent, commentary, case report, letter, or conference abstract; (2) not accessible in full text; or (3) unpublished.

### 2.3. Data Extraction

Following this screening and dereplicating process, 101 studies were deemed eligible and formed the basis for this narrative review. The selected literature was extracted for the following information, including the authors and the publication year, the sources of plasmalogens, the investigated molecular species, the methodology and approaches of investigation, and the key findings. This information was extracted and synthesized to present a coherent overview of the current state of knowledge, identify consensus and controversies in the literature, and highlight gaps for future research.

## 3. Structural Changes in Plasmalogens

### 3.1. Oxidation and Oxidative Degradation

Lipids are naturally susceptible to oxidation because of the double bonds in their fatty acyl chains, which serve as key sites for peroxidation under oxidative stress conditions. Various oxidizing agents have been shown to initiate plasmalogen oxidation both in vitro and in vivo. These include reactive oxygen species (ROS) such as peroxyl radicals, ultraviolet (UV) radiation, transition metal ions (e.g., Pb^2+^, Cu^2+^), which catalyze lipid peroxidation through Fenton-like reactions, and others [11,12,13,14]. Moreover, primarily owing to the unique sn-1 vinyl ether linkage, plasmalogens exhibit an even higher susceptibility to oxidation than other phospholipids, which can be explained as a lower bond dissociation energy and higher electron density of the vinyl ether bond than the ester bond or the saturated ether bond. Consequently, as the hydrogen atoms bonded to the carbon atom adjacent to the vinyl ether exhibit relatively low bond dissociation energies, the vinyl ether moiety, distinctively existing in plasmalogens, emerges as a significant target for ROS [15]. Using electron paramagnetic resonance oximetry and product analysis approaches, Broniec et al. revealed that the reaction of singlet oxygen with plasmalogens is significantly faster than other lipids, with the corresponding rate constants being one to two orders of magnitude higher [16].

#### 3.1.1. Oxidative Cleavage at the Vinyl Ether Bond of the sn-1 Position

**Figure 2:**
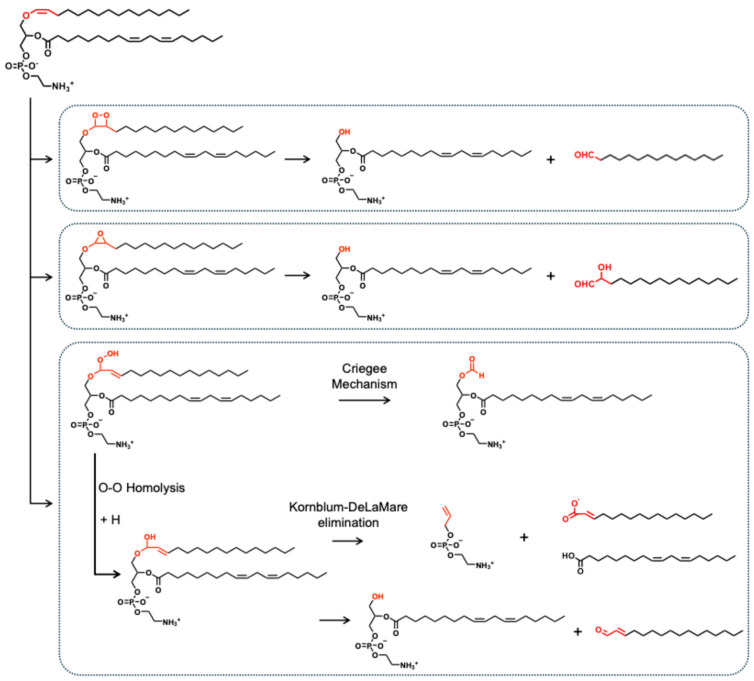
Oxidation routes of the sn-1 position in plasmalogens (taking PlsEtn p16:0/18:2 *n*-6 as an example), forming dioxetanes, epoxides, and hydroperoxides, respectively, together with further oxidation/degradation products (summarized based on references [17,18,19,20]).

Plasmalogen vinyl ether bonds are uniquely susceptible to oxidation, and different oxidants induce distinct cleavage pathways. Attack on the sn-1 vinyl ether yields unstable intermediates—usually three-membered epoxides, four-membered dioxetanes, or hydroperoxides (–OOH)—depending on the oxidant [17,18] (Figure 2). These intermediates eventually form lysophospholipids and small carbonyl compounds as products.

Singlet molecular oxygen (^1^O_2_), generated by UV light or photosensitizers, reacts with the vinyl ether bond to form a dioxetane ring. Such an intermediate undergoes a homolytic cleavage to yield a 2-lysophospholipid (for instance, PlsEtn → 2-lysophosphatidyl ethanolamine, 2-LPE; PlsCho → 2-lysophosphatidyl choline, 2-LPC), together with a truncated aldehyde (aldehyde is shortened by one carbon relative to the original sn-1 chain). As illustrated in Figure 1, ^1^O_2_ oxidation of PlsEtn p16:0/18:2 *n*-6 can result in 2-LPE 18:2 *n*-6 and pentadecanal (15:0 aldehyde). It was reported that 60 min of UV irradiation to PlsEtn generated aldehydes with odd-number carbon chains, such as 15:0, 17:0, and 17:1 [19]. It should be noted that such oxidation can occur without light, known as “dark photochemistry” of plasmalogens, according to Faria et al. [17]. In contrast, radical oxidants such as hydroxyl radicals (HO•) attack the vinyl ether bond to give an epoxy on the sn-1 position. Subsequently, this three-membered ring cleaves to form 2-lysophospholipid and α-hydroxyaldehyde. For example, Oxidation of PlsEtn p16:0/18:2 *n*-6 by radical oxidants can produce 2-LPE 18:2 *n*-6 and 2-hydroxyhexadecanal (16:0-OH aldehyde). Moreover, under the oxidation condition, the released aldehydes are unstable: they may rapidly oxidize to the corresponding fatty acids or react with amino groups on proteins/lipids to form Schiff-base adducts, as proposed by Stadelmann-Ingrand et al. [19]. Consequently, the dominant stable product observed from plasmalogen oxidation is usually the 2-lysophospholipid, with only transient truncated aldehydes and/or α-hydroxyaldehydes.

Plasmalogen oxidation can also occur through vinyl-ether hydroperoxide (–OOH) formation at the sn-1 position. Homolytic cleavage of the O–O bond produces an α,β-unsaturated hemiacetal, which then collapses to cleave off the alkyl chain and form 2-lysophospholipid again. Alternatively, vinyl ether hydroperoxides can undergo a Kornblum-DeLaMare elimination, during which both sn-1 and sn-2 chains of plasmalogens are removed, leaving only the glycerophospholipid backbone with a short enal (alk-1′-enyl) attached [20]. Using PlsEtn p16:0/18:2 *n*-6 as an example, the final oxidation products include prop-2-enyl phosphate ethanolamine, 2-ene-hexadecanoic acid, and linoleic acid, reflecting deep breakdown of the plasmalogen structure.

In addition to ring cleavage, an interesting rearrangement pathway can occur under certain oxidizing conditions, which yields a formyl group at the sn-1 position (e.g., PlsEtn p16:0/18:2 *n*-6 → PE 1:0/18:2). According to Khaselev et al., one possible mechanism for the formation of this product involves the migration of the vinyl group, a process recognized as a straightforward reaction of allylic hydroperoxides, referred to as the Hoch rearrangement. This is followed by the Criegee reaction, which involves the peroxyl rearrangement to form 1-formyl [20].

#### 3.1.2. Oxidation of Unsaturated Fatty Acyls in the sn-2 Position of Plasmalogens

**Figure 3:**
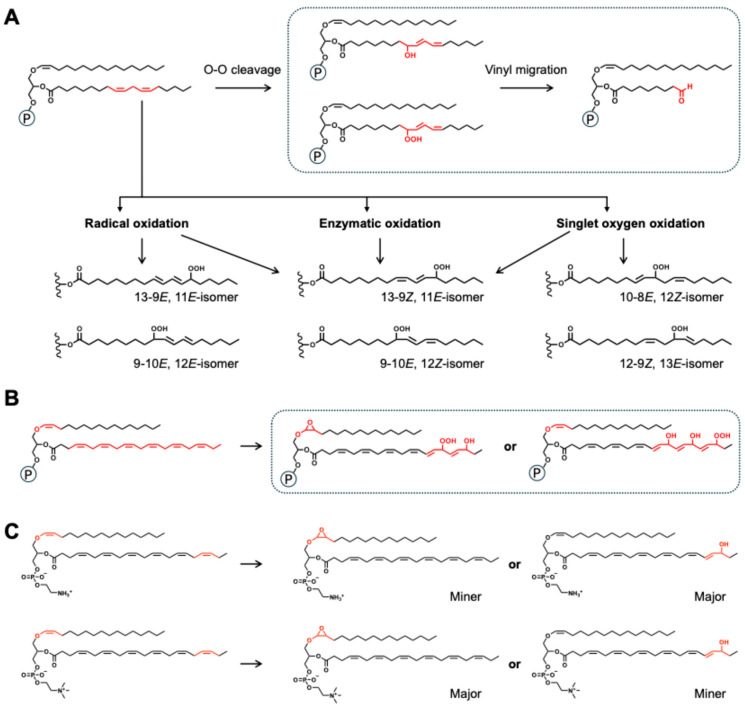
Oxidation routes of the sn-2 position in plasmalogens (summarized based on references [21,22]). (**A**) oxidative degradation of sn-2 fatty acyl chains via O-O cleavage, as well as radical, enzymatic, and singlet oxygen oxidation (taking Pls p16:0/18:2 *n*-6 as an example); (**B**) diversity of Pls + multiple [O] products, including Pls-epo, Pls-OH, and Pls-OOH, and their mixture (taking Pls p16:0/22:6 *n*-3 as an example); (**C**) differences between PlsEtn and PlsCho in selective oxidation in sn-1/sn-2.

Unsaturated fatty acyl chains at the sn-2 position of plasmalogens are prone to being attacked by oxidants through the same canonical pathways established for diacyl phospholipids (e.g., PE and PC), especially polyunsaturated fatty acyl (PUFA) chains, such as C20:4 *n*-6 (arachidonic acid, ARA), C20:5 *n*-3 (eicosapentaenoic acid, EPA), and C22:6 *n*-3 (docosahexaenoic acid, DHA). The most common oxidized molecules include hydroxides (Pls-OH) and hydroperoxides (Pls-OOH), often accompanied by double-bond migration or cis-trans isomerization. Various sources of oxidation, such as radical oxidation (including thermal and auto-oxidation), enzymatic oxidation (like lipoxygenase), and singlet oxygen oxidation (for example, photo-oxidation and inflammation), produce hydroperoxides (typically PUFA-OOH isomers) with distinct characteristics, as shown in Figure 3A.

Pls-OH or Pls-OOH can undergo further cleavage of the sn-2 acyl chain, producing shorter aldehyde-terminated fragments (Figure 3A). The length of these fragments depends on the unsaturated acyl residue and the –OH/–OOH position. Khaselev et al. investigated PlsCho p16:0/20:4 *n*-6 under peroxyl-radical stress generated by AAPH. They discovered that the dominant product was the C5 ω-aldehyde ester (PlsCho p16:0/5:0-al), indicating selective β-scission of the hydroperoxide at C-5 of ARA [20]. Moreover, Zemski et al. identified a series of PlsCho p16:0/22:6 oxidation products as sn-2 γ-hydroxy-α,β-unsaturated aldehydes, including PlsCho p16:0/4-hydroxy-7-oxo-hept-5-enoyl, PlsCho p16:0/7-hydroxy-10-oxo-dec-4,8-dienoyl, and PlsCho p16:0/10-hydroxy-13-oxo-tridec-4,7,11-trienoyl, using a MOX/BSTFA derivatization-LC-MS strategy, revealing the complexity of sn-2 acyls in plasmalogens [21].

Furthermore, Chen et al. investigated the oxidation of food-derived plasmalogens during long-term autooxidation and observed a more complex oxidation pattern, for which the multi-site oxidation of plasmalogens with up to four additional oxygen atoms attached to the intact plasmalogen molecules (for instance, from +1[O] to +4[O] for PlsEtn p16:0/22:6 *n*-3 and PlsCho p16:0/22:6 *n*-3) was discovered; these oxidation functional groups included epoxides, hydroxides, and hydroperoxides (Figure 3B) [22]. These results supported Zemski et al.’s findings that radical-induced peroxidation of plasmalogens leads to two main types of oxidation at the sn-1 and sn-2 positions, occurring together as a mixture [21]. Interestingly, the plasmalogen oxidation profile depended on the attached [O] number and the headgroup of plasmalogen. For the mono-oxygenated species (+1[O]), the oxidation products were either epoxides (Pls-epo, at the vinyl ether in the sn-1 chain) or hydroxides (Pls-OH, at the C=C bond in the sn-2 fatty acyl). Moreover, for PlsEtn, the primary oxidation products were PlsEtn-OH (abundance: PlsEtn p16:0-epo/22:6 < PlsEtn p16:0/22:6-OH), while PlsCho exhibited the opposite (e.g., PlsCho p16:0/22:6 was more likely to form PlsCho p16:0/22:6-OH than p16:0-epo/22:6) [22]. Whereas for di- to tetra-oxygenated species (+2[O] to +4[O]), hydroxides and hydroperoxides were scattered along the sn-2 chain, and there was no preference for epoxide/hydroxide/hydroperoxide in the oxidation products (Figure 3C) [22].

### 3.2. Enzymatic Degradation and Remodeling

**Figure 4:**
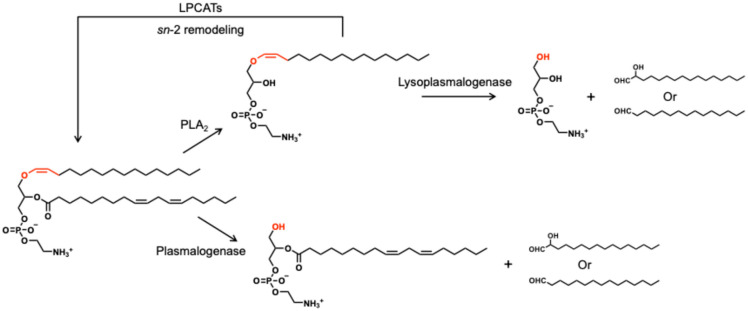
Enzymatic degrading and remodeling routes of plasmalogens (taking PlsEtn p16:0/18:2 *n*-6 as an example), forming characteristic aldehydes (summarized based on references [3,23,24,25,26,27,28,29,30]).

Plasmalogens are cleaved at the sn-1 vinyl ether linkage by a plasmalogen-selective phospholipase A_2_ (Pls-PLA_2_), releasing the sn-2 fatty acids and 1-O-alkenyl-2-lyso-sn-glycerophospholipids, i.e., lysoplasmalogens. Farooqui et al. demonstrated that this particular enzyme differed from cytosolic phospholipase A_2_ (cPLA_2_) and Ca^2+^-independent PLA_2_ (iPLA_2_) at molecular mass, substrate specificity, Km value, and other properties [23]. This enzyme was found enriched in neural membranes, and receptor-mediated activation of this enzyme has been implicated in plasmalogen breakdown during neurodegeneration and ischemia. It should be noted that such oxidation occurs even at 4 °C of refrigerated storage, resulting in plasmalogens as well as diacyl glycerophospholipids [24]. The yielded lysoplasmalogens undergo further hydrolysis by lysoplasmalogenase to form fatty aldehydes (or α-hydroxyaldehydes) and glycerol-phosphate headgroup moieties [3,25,26,27]. Alternatively, lysoplasmalogens can be re-acylated to regenerate the original plasmalogens or create new plasmalogen species with different sn-2 fatty acyl compositions [25,26,28], referred to as plasmalogen sn-2 remodeling (Figure 4). According to Thomas et al., the key enzymes of this remodeling process were lysophospholipid acyltransferases (LPLATs), specifically, lysophosphatidylcholine acyltransferase 3 (LPCAT3), which regulated the balance of 20:4 *n*-6 (ARA), 20:5 *n*-3 (EPA), and 22:4 *n*-6 acyls in plasmalogens, contributing to their sn-2 remodeling [29]. It is noted that the degradation process occurs during absorption and digestion. As Wang et al. demonstrated, EPA-enriched PlsEtn could be hydrolyzed to lysoPlsEtn with the release of EPA with the help of PLA2, during which the vinyl-ether bond at the sn-1 position was retained [30].

Besides the recycling pathway, plasmalogens can be directly degraded by plasmalogenase to generate 1-lysophospholipids and α-hydroxyaldehydes; in this route, the products are the same as those of the radical oxidant-induced oxidative degradation (Figure 4). All these findings imply that other radical sources (e.g., •OH, NO_2_•) should yield distinct aldehydic chain lengths and warrant systematic mapping of radical-specific “aldehyde fingerprints” as a characteristic composition of “plasmalogen-omics”. In addition, phospholipase C and D result in 1-alkenyl-2-lyso-sn-glycerol and 1-alkenyl-2-lyso-sn-glycerophosphate as degradation products, respectively [8,31], which function the same as other glycerophospholipids.

## 4. Variations in Plasmalogens

Beyond structural changes, plasmalogens also vary quantitatively in biological systems due to biosynthesis, degradation, remodeling, and certain external stressors. Not only concentrations, but also the composition of molecular species within the plasmalogen pool changes. These shifts often involve the shortening of sn-2 fatty acyl chains, the loss of PUFAs such as DHA and EPA, and the accumulation of oxidized, truncated, or degraded species, as a result of radical-mediated oxidation and/or enzymatic degradation (discussed in Section 3). In parallel, the balance between plasmalogens and diacyl analogs may also be disrupted. In conditions of plasmalogen depletion, a compensatory increase in diacyl phospholipids (typically PE and PC, corresponding to PlsEtn and PlsCho, respectively) has been observed, mediated by competition at phosphotransferases such as EPT and CPT [31,32]. In addition, the possible increase in lysophospholipids (typically LPE and LPC, corresponding to PlsEtn and PlsCho, respectively) may be attributed to the loss of intact plasmalogens during degradation, especially in the sn-1 position. These molecular-level transitions highlight the complexity of plasmalogen dynamics and emphasize the need for quantitative, species-specific analysis. Rather than simply measuring plasmalogen abundance as just a binary marker, recognizing its species-specific changes can provide deeper insights into disease progression, metabolic stress, and adaptive responses. All these variations are critical for understanding their role in health and disease, as well as their potential for clinical applications. Such variations are influenced by physiological conditions, pathological states, and nutritional supplementation.

### 4.1. Physiological and Clinical Parameters

Aging is one of the most significant physiological factors associated with a gradual decline in plasmalogen levels. Studies in healthy humans have revealed that plasmalogens (including PlsCho and PlsEtn) levels drastically decreased by approximately 40% in the elderly (≈70 years old) compared with mid-adulthood (≈30–40 years old), as reviewed previously [32]. Such a substantial reduction is thought to be linked to oxidative stress along with aging. This age-dependent decline is believed to stem from cumulative oxidative stress, reduced peroxisomal activity, and impaired enzymatic remodeling. On the other side, neonates have been found to have statistically significantly lower plasmalogen levels compared to older children [33]. Importantly, Beyene et al. revealed that, overall, men exhibit higher levels of ether-linked phospholipids than women. Specifically, for plasmalogens, there is a decline in PlsCho levels with age in both sexes. In contrast, PlsEtn displays a sex-divergent pattern: it decreases with age in men but increases in women. The authors suggest that these differences may be connected to menopause, which can influence neurodegenerative and cardiometabolic risks by affecting the metabolism of ether-phospholipids [34].

Other clinical parameters affect plasmalogen profile, such as body mass index (BMI). Higher BMI is associated with lower plasmalogen signatures [34], whereas Donovan et al. found the PlsEtn p18:0/20:4 *n*-6 as the distinguishing variable, which was significantly higher in the obese group (BMI: 49.87 ± 11.27) than the normal group (BMI: 25.76 ± 4.39) [35]. Some clinical indices also indicate plasmalogen changes; for instance, Maeba et al. demonstrated that serum plasmalogen levels positively correlated with high-density lipoprotein (HDL), but declined substantially with aging [36]. In addition, Mayneris-Perxachs et al. discovered that muscle carnosine level is known to be positively associated with plasmalogen level [37].

### 4.2. Genetic Diseases with Inherited Plasmalogen Deficiency

Barth syndrome is an X-linked potentially life-threatening recessive disease caused by mutations of a G4.5 gene in distal Xq28 [38], which encodes a mitochondrial transacylase named Tafazzin. Several studies have shown that plasmalogen decreases in Barth Syndrome. Kimura et al. found 35% decrease in PlsCho in mouse heart tissue, which accounted for around 11% in total lipid content (from 32.4% to 21.5%) [39]. In a similar study of multiple tafazzin knockdown mouse organs (brain, liver, and kidney), choline plasmalogen was dramatically reduced [40]. Consistently, for PlsEtn, the sharp reduction was detected in patient lymphoblasts [39,40].

Another rare inherited disorder, Rhizomelic Chondrodysplasia Punctata (RCDP), is also caused by impaired plasmalogen biosynthesis [41]. The severity of RCDP seems to be linked to the level of plasmalogens in fibroblasts from affected individuals [42]. PlsEtn decreased by 40% in non-severe cases, while in severe cases, the reduction exceeded 70% [42]. Therefore, it has been suggested that reduced plasmalogen levels may contribute to the symptoms observed in RCDP [41,43,44].

In addition, plasmalogen biosynthesis is profoundly impaired in peroxisome biogenesis disorders, such as Zellweger syndrome, leading to extremely low plasmalogen levels in multiple tissues [18].

### 4.3. Hepatic Metabolic Dysfunction

Endogenous hepatic plasmalogens are involved in fatty acid metabolism, and low plasmalogen levels contribute to severe hepatic dysfunction, fatty liver, non-alcoholic fatty liver disease (NAFLD)/nonalcoholic steatohepatitis (NASH), and even liver cirrhosis [45]. In a NASH model induced by cholesterol accumulation in the liver, plasmalogen levels decreased, thereby sensitizing animals to hepatocyte injury and NASH. Notably, DHA-containing PlsCho and PlsEtn decreased by over 20%, which was associated with reduced expression of Gnpat, the enzyme that limits plasmalogen biosynthesis [45]. Additionally, Wu et al. compared the lipidome in the liver and kidney of NASH model mice and found that PlsEtn (rather than PlsCho) containing PUFA species was significantly lower in the NASH group; these changes were more pronounced in the kidney than in the liver [46]. In clinical studies, circulating plasmalogens levels have also been found to be decreased in patients with NALFD/NASH [47,48]. Interestingly, lipidomics in NAFLD patients with the GG-genotype of PNPLA3, which is associated with a higher risk of advanced disease and fibrosis, showed even more reduced levels of total plasma plasmalogens compared to those with CC- and CG-allele [49].

The reduction in plasmalogens observed in the blood of NASH patients may result from oxidative stress damaging peroxisomes, which in turn impairs the biosynthesis of plasmalogens [50]. At the same time, plasmalogens have been explored as an indirect antioxidant via the Keap1-Nrf2 pathway against hepatic oxidative stress [51].

### 4.4. Neurodegenerative Diseases

Neurodegenerative disorders are involved in the deterioration of the brain due to the progressive loss of structure and/or function of neurons, for which a remarkable decrease in plasmalogen levels has been reported [52,53]. These molecular changes result in progressive memory loss along with mitochondrial dysfunction and oxidative stress and inflammatory damage to the [8,54].

Post-mortem analyses of the cortical tissues and cerebrospinal fluid from AD patients revealed a decrease in PlsEtn and PlsCho in both gray and white matter of their brains [55,56,57,58,59]. However, at the earlier stage of this disease, the loss of plasmalogen in the patients is more severe in white matter (40 mol%) than in gray matter (10 mol%) [54]. Kou et al. demonstrated that in the gray matter regions, peroxisomal parameters (e.g., peroxisomal density) in neurons become aggravated [59], which appeared together with synaptic loss, peroxisomal function impairment, and phospholipid (especially plasmalogens) alteration in AD. Moreover, some plasmalogen species, with DHA in particular, have been considered biomarkers for cognitive decline, being a hopeful diagnostic method for different stages of dementia [55,60,61,62,63]. However, Han demonstrated no correlation between low plasmalogen levels and ApoE4 (a biomarker of AD), questioning the link between plasmalogen loss and AD [64]. Interestingly, disease-associated plasmalogen loss is not always linear. Azad et al. observed an initial increase in PlsEtn levels in the hippocampus of AD model mice at early stages of pathology [65], potentially reflecting a transient compensatory mechanism.

Similarly, altered plasmalogen levels are examined in PD patients; for instance, PlsEtn decreased 30% in both plasma and erythrocytes [66]. Fabelo et al. proposed that plasmalogen loss at lipid domains from cortical gray matter led to impaired cellular signaling [67].

Multiple Sclerosis involves autoreactive lymphocyte invasion that causes localized inflammation, demyelination, axonal loss, and gliotic scarring, while mitochondrial dysfunction contributes to neuroinflammation [68,69]. The patients of both remission and relapse showed a marked decline of PlsCho and PlsEtn in serum [70], which is supposed to be the involved immune system stress-induced oxidation and the demyelination due to the myelin sheath membrane phospholipid decomposition [70]. However, Geroldinger-Simić et al. found that patients with systemic sclerosis showed a distinct shift: PlsCho species increased, while PlsEtn species decreased, particularly in those with concurrent lung fibrosis [71]. Therefore, the specific role of plasmalogens is yet to be studied.

### 4.5. Neurodevelopmental Disorders

Schizophrenia (SCZ) is a serious mental disorder affected by genetic and environmental risk factors, characterized by a variety of systemic abnormalities, such as abnormalities in neurodevelopment, inflammatory responses, and energy metabolism [72,73]. The phospholipid dissociation process of cell membranes was abnormal in SCZ patients [74]; particularly, the total PlsEtn concentration was significantly lower, showing its potential to be a biomarker as useful as other ether-phospholipids [74]. These results indicated that the dysregulation of membrane lipids. These findings supported the hypothesis that SCZ is a disorder of membrane lipid metabolism, and this abnormality of membrane lipid eventually affects neurological abnormalities and complex brain behaviors.

Autism manifests as a noticeable behavioral and developmental abnormality, while PlsEtn was decreased by 15% in the brains of autism rats [75]. Additionally, autistic patients showed a reduction of 15–20% in total plasmalogens according to two independent studies [76,77].

### 4.6. Cancers

Lee et al. conducted plasma plasmalogen profiling of different patients with cancers (liver, gastric, lung, colorectal, and thyroid). The results indicated that PlsEtn species (specifically, PlsEtn p16:0/20:4, p16:1/22:6, and p18:1/20:2) significantly decreased in liver, gastric, lung, and colorectal cancers but were high in thyroid cancer. Moreover, PlsEtn p18:0/20:4, p18:1/20:4, and p18:1/20:5 decreased in liver, lung, and colorectal cancers [78]. Another study on hepatocellular carcinoma reported that PE and PlsEtn species were significantly reduced in serum samples of the patients, in which two PlsEtn species, p16:0/20:4 and p18:1/22:5, were supposed to be useful biomarkers [79].

### 4.7. Infectious Diseases

Since plasmalogens are crucial for membrane dynamics, act as antioxidants, and serve as precursors for other bioactive lipids, they play a key role in inflammation related to disease and infection. Recent studies have exhibited that plasma plasmalogen levels are reduced in humans with severe COVID-19 [80,81,82]. As a primary pool of PUFAs, plasmalogens (especially those with PUFA at the sn-2 position) participate in both immune and structural cells; therefore, infections like COVID-19 stimulate plasmalogens, generating lipid mediators and thus alter the plasmalogen profile.

### 4.8. Lifestyles

Despite physiological and pathological factors, unhealthy lifestyles contribute to circulating plasmalogen loss. Smoking and alcohol drinking, as popular unhealthy habits and risky factors of various chronic diseases, negatively affect plasmalogen levels. Beyene et al. developed a plasmalogen score using the compositional data of all PE and PlsEtn species in plasma, which can be associated with metabolic risk factors. They found the strongest negatively correlated factor to be alcohol intake [83]. While Middlekauff et al. investigated the influence of smoking on the plasma lipidome and discovered that both tobacco and electronic cigarettes result in depletion of PlsEtn (typically, p16:0/18:2 and p18:1/18:2), and intriguingly, females suffer more severely than males [84]. In addition, diets with excessive fats, lacking essential fatty acids, or containing high levels of oxidants may compromise plasmalogen homeostasis.

## 5. Exogenous Sources of Plasmalogens for Nutritional Supplements

Exogenous plasmalogens from specific dietary sources have gained increasing attention. Therefore, recent research focuses on plasmalogen content in food sources, its nutritional implications, and how these dietary sources may influence health. Naturally, plasmalogens are primarily those derived from animal products, such as fish, meat, and dairy products, as well as certain marine foods [1]. Studies have revealed that marine animals, especially fish and mollusks (shellfish), are particularly abundant in plasmalogens. For instance, shark liver oil and some fish oils have been reported to contain high levels of plasmalogens, particularly PlsEtn with PUFAs [85], making them potential nutritional supplements protecting against metabolic dyslipidemia and inflammation. Importantly, the exact content of plasmalogens in these foods can vary depending on the species and the specific tissue, such as liver, muscle, or brain. Therefore, certain parts of foods, often underused in diets due to cultural or regional preferences, may serve as cost-effective sources of plasmalogens.

For instance, porcine brain has been identified as a promising source of plasmalogens. A recent study by Wu et al. determined plasmalogen levels in porcine brain tissues and revealed substantial levels of PlsEtn rich in 18:1, 20:4, 22:4, and 22:6 as fatty acyls, which are characteristic of brain lipids [86]. The study demonstrated the feasibility of using porcine byproducts as an economical source of nutraceuticals. Similarly, Yunoki et al. reported that chicken skin contains considerable levels of plasmalogens (predominantly PlsEtn) and considered it a valuable source of functional ether lipids for dietary supplementation [87]. Meanwhile, Mawatari et al. showed that chicken skin-derived plasmalogens could serve as nutritional supplements and increase the relative content of erythrocyte membrane plasmalogens (mainly PlsEtn) in rats [88]. Another promising byproduct, scallop viscera, shows potential as a natural plasmalogen source. Although it is unsuitable for eating due to various hazards, it contains comparable PlsEtn content to muscle and mantle, and provides even a higher PlsEtn to PE ratio [89]. As interest in utilizing these byproducts for functional food applications grows, microorganism-derived plasmalogens are attracting researchers’ attention, representing a new way of effectively utilizing bacterial resources as a “food” source. As Sato et al. reported, *Selenomonas ruminantium*-derived PlsEtn mainly consists of p16:1/14:0 (68.4%) and p16:1/16:1 (29.2%), which appear uncommon compared with those from mammals [90]. In addition, plasmalogen biosynthesis in Bacteria has been explored and even optimized (e.g., strain selection and culturing conditions), as seen in *Clostridium perfringens* and *C. pasteurianum* [91,92]. These findings expand natural sources and scalable production routes for plasmalogens, contributing not only to the circular economy in food production but also aligning with high-value food development principles focused on nutrition.

Regarding the use of nutritional supplements as a treatment for plasmalogen deficiency, plasmalogen replacement therapy (PRT) has been shown to effectively restore plasmalogen levels and improve disease symptoms in various clinical applications [32]. Although plasmalogens may be hydrolyzed during digestion, PRT aims to replace the biomembrane phospholipid and correct plasmalogen deficiency, and it is administered orally, which is a key advantage of this therapy. The compounds provided can be plasmalogens (either PlsCho and PlsEtn, extracted from natural sources or synthesized) or plasmalogen precursors (e.g., alkylglycerols), with shark liver oil and other marine products being common sources [83,85,93]. Many studies have demonstrated a universal increase (i.e., recovery from deficient levels) in all plasmalogens, including both PlsCho and PlsEtn, in a dose-dependent manner in clinical trials as well as animal studies [83,85,94,95,96,97]. This direct strategy of plasmalogen supplementation holds promising potential, positioning it as a new nutrient that can significantly benefit health and well-being.

## 6. Limitations, Challenges, and Perspectives

While plasmalogens are known to play a crucial role in various physiological and pathological conditions, our understanding of how they change with aging and disease is still primarily based on cross-sectional data. To gain a better understanding of the underlying mechanisms, we need more longitudinal, pathway-specific evidence to distinguish the effects of oxidative stress, peroxisomal dysfunction, and enzymatic remodeling.

One of the solution factors should be the analytical approach. Despite significant progress in LC-MS-based lipidomics, current plasmalogen analyses still encounter practical bottlenecks during the pre-analytical and cleanup stages: sample handling easily causes artifactual oxidation and quantitation drift, and workflows are not yet standardized across laboratories, which limits comparability [98,99]. Although progress has been made in distinguishing isomers, for example, in separating plasmenyl (with –C–O–C=C– linkage) and plasmanyl (with –C–O–C–C– linkage) phospholipid species using LC-MS [100], more universal, practical, and robust methods often require specific chromatographic conditions or derivatizations (pre-column reactions). As a result, they remain challenging to implement routinely in high-throughput settings [101]. In particular, direct monitoring of vinyl-ether requires specialized chemical modifications (e.g., labeling with fluorescence or isotopes), which have not yet been widely adopted in the population or clinical pipelines. These strategies may contribute to clarifying the absorption, digestion, metabolism, and excretion of plasmalogens. Furthermore, advanced analyses can contribute to identifying, screening, and expanding food sources rich in certain beneficial plasmalogen species, as well as mapping how processing and storage affect their changing profiles and bioavailability.

In conclusion, while a general decline in plasmalogen levels is observed across aging and disease, the patterns of variation often involve specific subclasses and molecular species rather than uniform depletion. These changes indicate selective vulnerability, enzymatic remodeling, or compensatory lipid shifts. Therefore, a better understanding of plasmalogen changes is desired to bridge chemical diversities with nutritional implications, thereby advancing both mechanistic insights and health-promoting applications.

## Data Availability

Not applicable.

## Abbreviations

AD	Alzheimer’s disease
ARA	Arachidonic acid
CPT	Choline phosphotransferase
DHA	Docosahexaenoic acid
DHAP	Dihydroxyacetone phosphate
EPA	Eicosapentaenoic acid
EPT	Ethanolamine phosphotransferase
ER	Endoplasmic reticulum
G3P	Glycerol-3-phosphate
GPC	Glycerophosphocholine
GPE	Glycerophosphoethanolamine
HDL	High-density lipoprotein
HPLC	High-performance liquid chromatography
HR-MS	High-resolution mass spectrometry
LPC	Lysophosphatidyl choline
LPE	Lysophosphatidyl ethanolamine
LPCAT	Lysophosphatidylcholine acyltransferase
LPLAT	Lysophospholipid acyltransferases
MS	Mass spectrometry
NAFLD	Non-alcoholic fatty liver disease
NASH	Nonalcoholic steatohepatitis
PC	Phosphatidylcholine
PD	Parkinson’s disease
PE	Phosphatidylethanolamine
PEMT	Phosphatidylethanolamine *N*-methyltransferase
PLA	Phospholipase A
PLC	Phospholipase C
PlsCho	Plasmalogen choline
PlsEtn	Plasmalogen ethanolamine
PRT	Plasmalogen replacement therapy
PUFA	Polyunsaturated fatty acyl or polyunsaturated fatty acid
RCDP	Rhizomelic Chondrodysplasia Punctata
ROS	Reactive oxygen species
SCZ	Schizophrenia
UV	Ultraviolet
